# Omadacycline for peritoneal dialysis-associated peritonitis caused by *Coxiella burnetii*: a case report and literature review

**DOI:** 10.3389/fmed.2026.1829483

**Published:** 2026-07-14

**Authors:** Xiaobing Hong, Zinan Cai, Zelin Yu, Hongbo Fu, Jianpeng Cai, Zhijian Wu, Xiuming Wu, Zejian Kuang

**Affiliations:** 1 Department of Pharmacy, The Second Affiliated Hospital, Shantou University Medical College, Shantou, China; 2 Department of Critical Care Medicine, The Second Affiliated Hospital, Shantou University Medical College, Shantou, China

**Keywords:** *Coxiella burnetii*, metagenomic next-generation sequencing (mNGS), omadacycline, peritoneal dialysis-associated peritonitis, Q fever

## Abstract

**Introduction:**

Peritoneal dialysis-associated peritonitis (PDAP) is a serious complication of peritoneal dialysis (PD), contributing significantly to hospitalization rates and mortality. In recent years, infections caused by uncommon pathogens such as *Coxiella burnetii* have increasingly been identified, posing significant challenges to managing PDAP.

**Case presentation:**

We report a 62-year-old male hospitalized for recurrent PDAP unresponsive to empirical antibiotics (meropenem, later meropenem/vancomycin). Metagenomic next-generation sequencing (mNGS) of peritoneal fluid identified *C. burnetii*. Intravenous omadacycline was initiated as part of a multi-agent regimen (100 mg daily after 200 mg loading dose). Within 48 h, hemodynamic stability was achieved, and inflammatory markers (procalcitonin, C-reactive protein, effluent white blood cell count) normalized progressively over the subsequent week. The patient recovered fully and was discharged, and remained relapse-free during 3 months of follow-up.

**Conclusion:**

This case highlights the critical importance of identifying pathogens in patients with PDAP. Despite significant confounders (concurrent broad-spectrum antibiotics, ICU support, and polymicrobial infection) that limit definitive attribution, the use of omadacycline was associated with clinical recovery and suggests a potential role as an alternative therapeutic option for *Coxiella burnetii* infection. Further studies are warranted to validate its efficacy.

## Introduction

Peritoneal dialysis (PD) is a primary renal replacement therapy for patients with end-stage renal disease. PD shows advantages such as preservation of residual renal function, lower treatment costs, and greater convenience for self-management, when comparing to hemodialysis (1), thereby improving quality of life. However, peritoneal dialysis-associated peritonitis (PDAP) remains a major complication, potentially leading to treatment failure, increased hospitalization rates, and increased mortality, accounting for approximately 15% of PD patients death (2).

The main pathogens causing PDAP are Gram-positive bacteria (60%–80% of cases), including *Staphylococcus epidermidis*, *Staphylococcus aureus*, and *Streptococcus* species, followed by Gram-negative bacteria (15%–30%) with common isolates such as *Escherichia coli*, *Klebsiella pneumoniae*, and *Pseudomonas aeruginosa*. While fungal peritonitis account for 3%–5% of cases, and culture-negative peritonitis (10%–20%) remains a significant clinical concern (3). Recent advances in diagnostic techniques and evolving host immune status have revealed an increasing incidence of infections caused by rare pathogens, such as *Coxiella burnetii* (4–6). Diagnosing *C. burnetii* poses challenges due to its atypical clinical presentation and suboptimal diagnostic yield with conventional culture methods, often resulting in delays in diagnosis and treatment (7).

*Coxiella burnetii* infection is an intracellular microorganism responsible for a zoonosis known as Q fever. Standard therapy typically involves doxycycline as the first-line agent, sometimes used in combination with hydroxychloroquine. However, this regimen is complicated by potential adverse effects (including severe allergic reactions) (8), emerging doxycycline-resistance, and potential drug-drug incompatibilities (9, 10). These limitations underscore the critical need to explore alternative therapeutic strategies to improve clinical outcomes in Q fever patients.

We herein present a case of PDAP caused by *C. burnetii*, successfully treated with omadacycline. This report also reviews the epidemiology, diagnostic approaches, and pharmacotherapeutic considerations for *C. burnetii* infections to inform clinical decision-making.

## Case presentation

### Patient history and admission

A 62-year-old male with hypertension, coronary heart disease, and Parkinson’s disease was hospitalized for peritoneal dialysis-associated peritonitis (PDAP). Seven years earlier, he initiated peritoneal dialysis (PD) following a diagnosis of chronic kidney disease stage 5 due to elevated serum creatinine. The patient had a history of multiple PDAP-related hospitalizations. On March 21, 2025, he was hospitalized in Guangzhou and Shantou for abdominal pain and turbid peritoneal effluent. Although initial treatment yielded some improvement, symptoms recurred and persisted. He was then admitted to our nephrology department on April 26, 2025, with recurrent abdominal pain, turbid effluent, chills, and anorexia. The patient was a long-term resident of Guangdong Province without known exposure to cattle or goats. Prior to admission, his PD exchanges were primarily performed by his spouse. Notably, his underlying Parkinson’s disease caused hand tremors and motor rigidity, which likely compromised his ability to maintain strict aseptic technique during the procedure, representing a significant risk factor for recurrent PDAP.

### Admission examination

On admission, vital signs were recorded [Heart rate, 126 bpm; Temperature, 36.4 °C; Blood pressure, 91/63 mmHg (without antihypertensive medication)]. The patient was alert but lethargic, with reduced verbal output. Respiratory examination showed clear lung fields bilaterally without rales. Abdomen examination showed distension with generalized tenderness and involuntary guarding, though no rebound tenderness was presented. The PD catheter exit site in the right lower quadrant was clean and dry. No lower extremity edema was observed.

### Initial investigations

**FIGURE 1:**
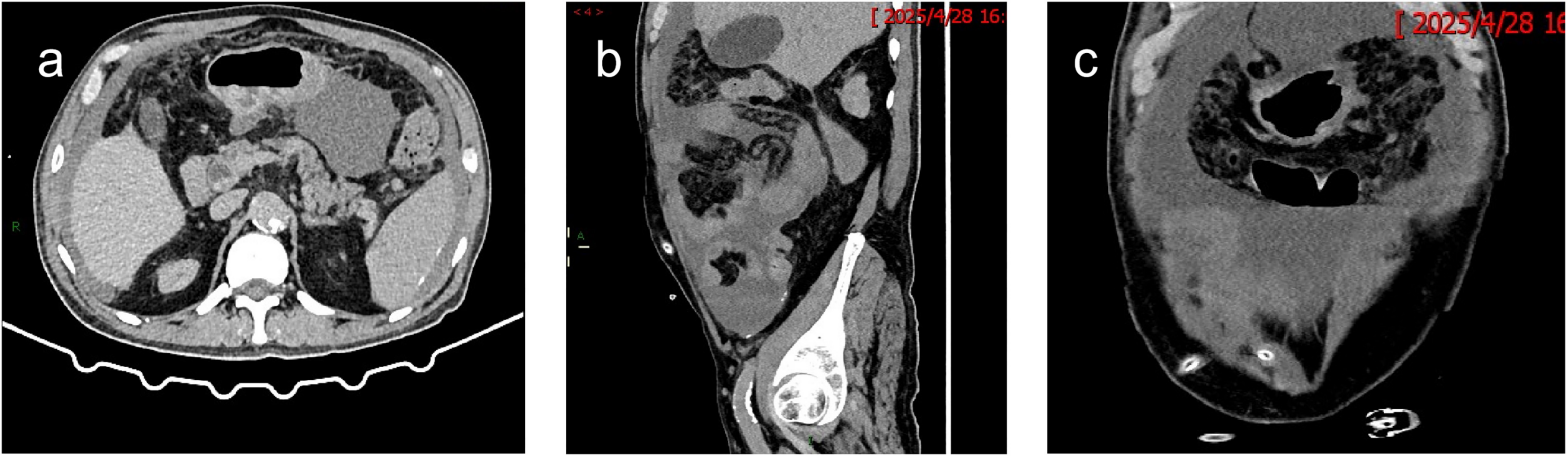
CT of the abdomen on the axial plane **(a)**, the sagittal plane **(b)**, and the coronal plane **(c)**.

Peritoneal fluid analysis showed marked leukocytosis (WBC 17,357 × 10^6^/L) with 93.4% polymorphonuclear cells; peritoneal fluid cultures remained negative. Blood tests demonstrated a normal white blood cell count (WBC 4.6 × 10^9^/L) with a mildly elevated neutrophil percentage (77.4%), hypoalbuminemia (28.4 g/L), elevated creatinine (761 μmol/L), and significantly increased procalcitonin (PCT 3.75 ng/mL). The result of chest radiograph showed increased bilateral pulmonary inflammatory infiltrates, while abdominal CT confirmed peritoneal thickening consistent with peritonitis (Figure 1).

### Hospital course and therapeutic interventions

Given the history of recurrent PDAP, empirical intraperitoneal antibiotic therapy with meropenem (1 g twice daily) was initiated. By post-admission day 7 (May 3), repeat testing showed decreased inflammatory markers (blood PCT 1.72 ng/mL; effluent WBC 3,796 × 10^6^/L) despite persistent clinical symptoms. On day 8 (May 4), a venous catheter was placed for continuous renal replacement therapy (CRRT) after the patient declined recommended PD catheter removal. The patient subsequently deteriorated precipitously by day 12 (May 8). He developed septic shock (BP 90/60 mmHg, fever) with neurological decline (obtunded, disoriented, and drowsiness) and respiratory symptoms (cough, chest tightness). Laboratory findings showed increased leukocytes (WBC 23.8 × 10^9^/L), increased neutrophil percentage (93.8%), anemia (Hb 97 g/L), thrombocytopenia (platelets 90 × 10^9^/L), and markedly elevated inflammatory markers (PCT 6.65 ng/mL; CRP 6.33 mg/dL), necessitating norepinephrine infusion and ICU transfer.

Antibiotic therapy was then escalated to intravenous meropenem (1 g every 6 h) and intensified intraperitoneal regimen (meropenem 1 g twice daily plus vancomycin 0.2 g twice daily). Peritoneal fluid was concurrently sent for conventional culture and metagenomic next-generation sequencing (mNGS). On hospital day 15 (May 11), mNGS identified *Coxiella burnetiid* (2 specific reads, 0.009% relative abundance; total sequencing depth 13,246,978 reads; Q30 91.28%). This finding was corroborated by subsequent clinical history revealing regular consumption of raw goat milk over the preceding 6 months. Given high suspicion for concomitant *C. burnetii* infection, intravenous omadacycline tosylate was added to the ongoing regimen of intravenous and intraperitoneal meropenem and intraperitoneal vancomycin on day 16 (May 12), with a 200 mg loading dose followed by 100 mg once daily maintenance therapy.

### Outcome and follow-up

**FIGURE 2:**
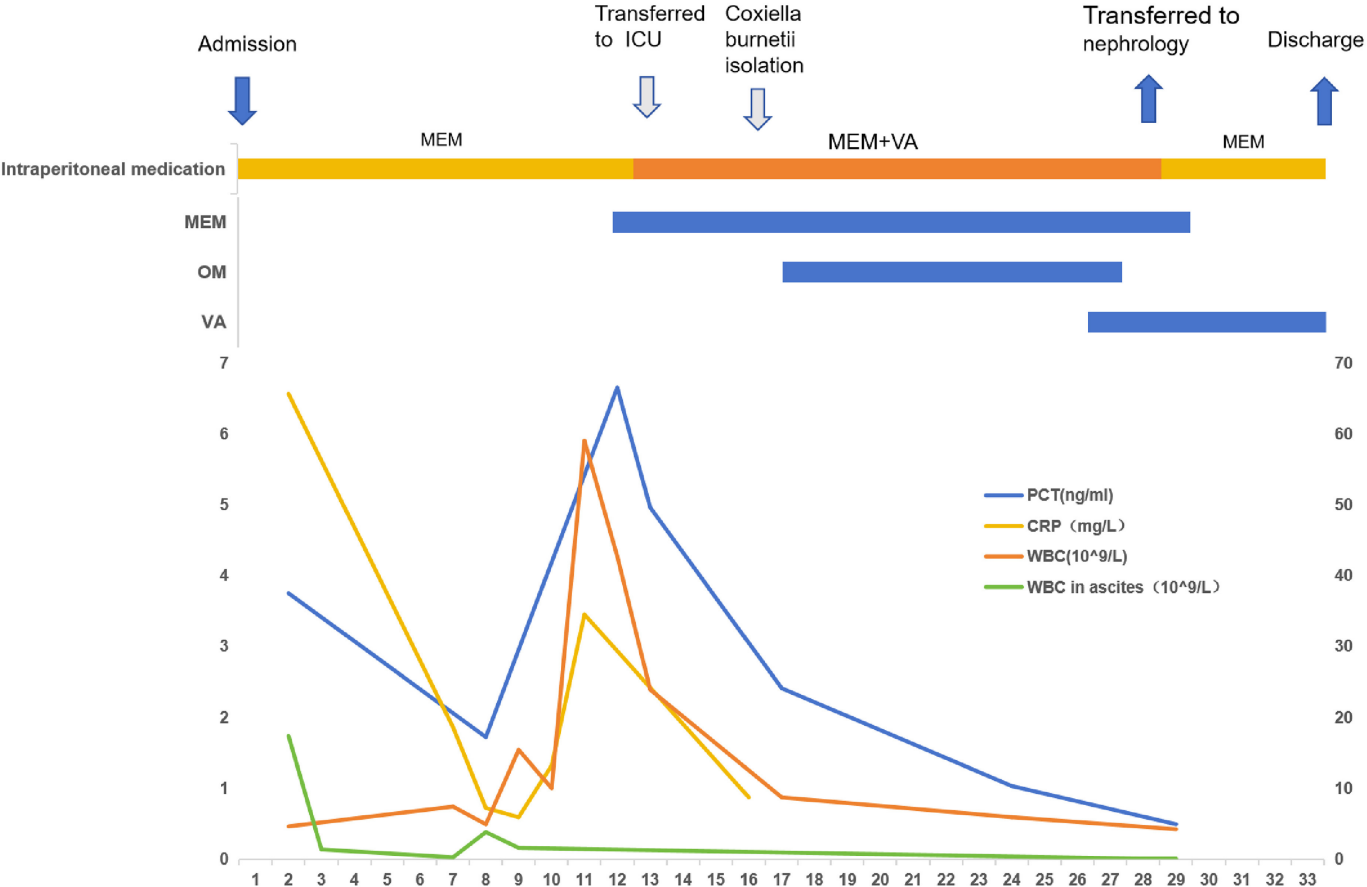
Inflammatory indicators and antibiotic use during hospitalization. CRP, C-reactive protein; ESR, erythrocyte sedimentation rate; WBC, white blood cell; MEN, meropenem; OM, omadacycline; VAN, vancomycin.

Following omadacycline initiation on day 16, rapid clinical improvement was observed. By May 14 (day 18), the patient achieved normothermia and hemodynamic stability, allowing discontinuation of norepinephrine. Repeat peritoneal fluid analysis showed effluent with 53.8% polymorphonuclear cells and decreased WBC (0.00 × 10^6^/L). Subsequent laboratory monitoring revealed progressive normalization of inflammatory markers. By May 19 (day 23), blood tests demonstrated WBC 5.9 × 10^9^/L, Neutrophils 65.7%, PCT 1.03 ng/mL, and CRP 0.87 mg/dL, enabling cessation of intravenous meropenem. Repeat blood and peritoneal fluid mNGS testing on May 21 (day 25) detected *Enterococcus faecium* without evidence of *C. burnetii*. Considering the patient’s multiple comorbidities and immunosuppression state, intravenous vancomycin (0.5 g every 8 h) was initiated while omadacycline was discontinued. Subsequent inflammatory markers and peritoneal fluid parameters normalized. The patient was transferred back to the nephrology ward on May 23 (day 27) and discharged fully recovered on May 28 (day 32). At 3 month post discharge follow-up, the patient had no recurrence of symptoms or signs of peritonitis. The timeline of antimicrobial therapy and inflammatory markers are shown in Figure 2.

## Discussion and conclusion

*Coxiella burnetii* is an obligate intracellular Gram-negative coccobacillus with multiple animal hosts, including sheep, cattle, and goats. Humans infection typically occurs through inhalation of contaminated aerosols, contacting with body fluids from infected animals, or less commonly, ingestion of unpasteurized dairy products (11). Although only 1%–5% of acute Q fever patients progress to chronic disease, this occurs more frequently in individuals who are immunocompromised or have underlying cardiac or vascular conditions (12). A French review of 84 chronic Q fever cases revealed that 20% involved immunosuppression patients, including those with conditions such as cancer, renal transplantation, chronic myeloid leukemia, corticosteroid therapy, acquired immunodeficiency syndrome, postpartum status, chronic alcoholism, and renal dialysis (13). Our patient, who has undergone peritoneal dialysis for 7 years, exemplifies this risk profile, since chronic exposure of peritoneal cells to dialysate may alter normal immune responses to bacteria, such as by diminishing bacterial opsonization (14). The most common chronic manifestations of Q fever are infective endocarditis, chronic vascular infections, and bone and joint infections, which also represent the predominant forms reported in case reports (15–17). *Coxiella burnetii*-induced peritonitis associated with peritoneal dialysis remains is extremely rare, with only three such cases documented globally to date (see Table 1) (4–6). The epidemiology of these infections aligns with classic Q fever transmission patterns, where typical sources include livestock exposure (e.g., sheep or cattle) or consumption of contaminated dairy products. This correlation was observed in our patient, who had consumed goat’s milk before symptom onset. Nevertheless, approximately 30% of Q fever cases lack identifiable exposure history.

**TABLE 1:** Clinical factors, treatment details, and outcomes among 3 patients caused by *Coxiella burnetii (C. burnetii)* peritonitis.

Author, year	Host factors	Methodology	Prior antimicrobial therapy	Treatment	Outcome, follow-up duration
(6)	A 45 y/o woman, hypertension, undergone PD for 5 years due to end-stage renal disease engaged in cattle and sheep breeding	metagenomic next-generation sequencing	vancomycin, etimicin, piperacillin	About 12 weeks of doxycycline (100 mg twice daily) and moxifloxacin (400 mg once daily) orally	The WBC of the PD effluent decreased to within the normal range and the culture of PD effluent was negative for *C. burnetii* at 4 months after discharge.
(4)	A 55 y/o man, with type 2 diabetes mellitus, sheep breeding in his neighborhood	Serological survey for *C. Burnetii*	NS	Two weeks of doxycycline (100 mg twice daily) and followed by 14 days of levofloxacin (500 mg/day) orally	The symptoms resolved completely without any sequelae.
(5)	A 41 y/o man, peritoneal dialysis for 6 years due to hypertensive nephropathy living in a village with sheep and goats	Serological survey for *C. burnetii*	Tazobactam-piperacillin vancomycin	10 days of doxycycline and ciprofloxacin, followed by 5 weeks of doxycycline, ciprofloxacin, and rifampicin	Phase 2 IgG titer decreased to a titer of 1/256, and the cell counts in the peritoneal fluid and ascites and thickening of the peritoneum resolved

y/o = year old; NS = not specified;

Diagnosing Q fever poses significant challenges because *Coxiella burnetii* is an obligate intracellular pathogen, which renders routine ascitic fluid cultures virtually sterile. This diagnostic limitation was exemplified in our case, where empirical antibiotics therapy with meropenem and vancomycin failed despite transient biomarker improvement, while cultures remained persistently negative until metagenomic next-generation sequencing (mNGS) detected the pathogen. Non-specific manifestations such as persistent low-grade fever, abdominal pain, and elevated inflammatory markers further complicates recognition, as these features may mimic more common conditions like *Enterococcus faecium* co-infection or culture-negative peritonitis. Initial transient improvement in PCT with broad-spectrum antibiotics (meropenem/vancomycin) obscured the diagnosis in the present case, preceding a dramatic clinical and biochemical deterioration signifying treatment failure. Although serology demonstrating Phase II IgG titers ≥1:128 remains the diagnostic gold standard, ascitic fluid mNGS played a pivotal role in this case despite the low read counts, guiding initial targeted therapy when conventional cultures failed. This underscores molecular diagnostics’ critical value for identifying elusive pathogens when conventional methods fail. Given the diagnostic complexities, extended and pathogen-targeted antibiotic therapy becomes imperative. Current guidelines, including those from the U.S. CDC (2013) and British Society for Antimicrobial Chemotherapy, recommend ≥18 months of combination therapy of doxycycline plus hydroxychloroquine or ciprofloxacin for chronic Q fever (18). For doxycycline intolerance, alternatives such as minocycline, clarithromycin, fluoroquinolones, TMP/SMZ, or rifampin may be considered according to established references including the *ABX Guide*, *Sanford Guide* (16). Notably, emerging reports of rising doxycycline MIC values suggest developing resistance (8–10).

It is crucial to acknowledge the significant confounders in attributing the clinical response solely to this agent. The patient’s improvement coincided with multiple major interventions. First, broad-spectrum antibiotics (intravenous and intraperitoneal meropenem, intraperitoneal vancomycin) were continued after omadacycline initiation. While these agents lack intracellular activity against *C. burnetii*, they may have contributed to overall infection control, particularly against coexisting pathogens. Second, the patient received ICU-level supportive care including vasopressors and CRRT, which could have contributed to hemodynamic stabilization and clearance of inflammatory mediators. Third, repeat mNGS of peritoneal fluid subsequently detected *Enterococcus faecium*-a vancomycin-susceptible organism. Intraperitoneal vancomycin was initiated prior to omadacycline; therefore, the observed improvement may be partially attributable to vancomycin activity against *E. faecium* rather than omadacycline against *C. burnetiid*, The antibiotic susceptibility profiles of these two pathogens are summarized in Table 2. The possibility of polymicrobial infection cannot be excluded. Given these confounders, this case provides no reliable evidence for the independent efficacy of omadacycline; at most, it demonstrates feasibility of its use as part of a multi-agent regimen.

**TABLE 2:** Summary of antibiotic susceptibility profiles of *Coxiella burnetii* and *Enterococcus faecium.*

Pathogen	Typical susceptibility	Typical resistance
*Coxiella burnetii*	Doxycycline, omadacycline, other tetracyclines, hydroxychloroquine (adjunct)	Beta-lactams, glycopeptides, aminoglycosides (variable)
*Enterococcus faecium* (VSE)	Vancomycin, ampicillin (if susceptible), linezolid, daptomycin	Often resistant to quinolones, aminoglycosides (low level)

The definitive diagnosis of Q fever and the differentiation between acute and chronic phases rely on serological criteria (Phase II IgG ≥ 1:128 for acute; Phase I IgG ≥ 1:800 for chronic). A major limitation of this case is the absence of serological confirmation. Consequently, the diagnosis heavily relied on mNGS. While the read count was low (2 specific reads), we considered it clinically significant rather than a background artifact. This is because *C. burnetii* is neither a commensal organism nor a typical environmental/reagent contaminant; thus, even low-level detection in a sterile site carries high specificity. Furthermore, this low yield is pathophysiologically attributable to two factors: its obligate intracellular nature within macrophages limits the release of free DNA into the effluent, and the continuous exchange of peritoneal dialysis fluid significantly dilutes residual pathogen nucleic acids. Based on the acute presentation (septic shock, rapid resolution) and absence of stigmata of chronic Q fever (e.g., endocarditis, hepatitis), we classified this as presumptive acute Q fever.

For acute Q fever, the recommended duration of doxycycline is 14 days. Our patient received only 10 days of intravenous omadacycline, which is shorter than the standard of care. Despite this, the patient achieved complete clinical recovery and remained relapse-free during 3 months of follow-up. This outcome may be explained by omadacycline’s long half-life and potent intracellular activity (19, 20), but we emphasize that this single case does not support shortening the standard 14-day course. Clinicians should adhere to established guidelines, and our deviation from them is a management limitation that we acknowledge. In conclusion, this case highlights the diagnostic challenges of rare PDAP pathogens and the difficulty of attributing outcomes in critically ill patients receiving multiple concurrent interventions. Omadacycline may be considered as an adjunctive option for suspected *C. burnetii* infection, but its independent efficacy remains unproven.

## Conclusion

*Coxiella burnetii* is a rare but important cause of PDAP, posing significant diagnostic challenges due to negative cultures by conventional culture methods and non-specific presentation. This case demonstrates that mNGS is a powerful tool for the rapid identification of such fastidious pathogens in critically ill PDAP patients, especially when combined with detailed epidemiological history. Furthermore, it provides preliminary clinical evidence for the potential role of omadacycline as an adjunctive therapeutic option for *C. burnetii* infection, although confounding factors such as concurrent antibiotics and polymicrobial infection limit definitive attribution of clinical response. Omadacycline warrants further investigation as a valuable alternative for the treatment of Q fever, especially in cases of intolerance or resistance to first-line agents.

## Data Availability

The raw data supporting the conclusions of this article will be made available by the authors, without undue reservation.
